# Expansion and characterization of tumor-infiltrating lymphocytes from human sarcoma

**DOI:** 10.1186/2051-1426-3-S2-P19

**Published:** 2015-11-04

**Authors:** MacLean Hall, John Mullinax, Erica Royster, Angela Reagan, Ricardo Gonzalez, Jose Pimiento, Shari Pilon-Thomas, Amod Sarnaik

**Affiliations:** 1 H. Lee Moffitt Cancer Center, Tampa, FL, USA; 2 H. Lee Moffitt Cancer Center & Research Institute, Tampa, FL, USA

## Background

Adoptive Cell Transfer (ACT) using Tumor Infiltrating Lymphocytes (TIL) has previously been shown at our institution and others to be an effective treatment for metastatic melanoma, resulting in a 38% response rate [1]. We applied this therapy to other solid tumors that have demonstrated a positive correlation between immune infiltrates and patient outcome [2]. Specifically in sarcoma, intra-tumoral CD4+ and CD8+ T cells have been detected, but their potential for *ex vivo* expansion is still relatively unexplored [3]. In this study, we investigated the feasibility of expanding TIL from surgically resected sarcoma specimens and analyzed the phenotype of these lymphocytes.

## Methods

Four different subtypes of sarcoma were surgically resected from patients accrued under an IRB approved research protocol (MCC50064). A portion of the tumor specimens was digested and immediately phenotyped by flow cytometry. The remaining tumor was minced and plated as fragments for the isolation of TIL, which were expanded *in vitro* for six weeks using high dose IL-2. Eight separate TIL cultures were established and phenotyped by flow cytometry.

## Results

Analysis of enzymatically digested human sarcoma specimens showed that 64% of lymphocytic infiltrates were CD3+ cells. TIL were isolated from fragments of each of the four sarcoma specimens as eight individual cultures and propagated *in vitro*, with TIL observed in 59 out of 84 (70%) fragments. Of the expanded CD3+ TIL, on average 39% were CD8+ T cells that expressed both the co-stimulatory molecule 4-1BB (33%) and inhibitory PD-1 (41%) by flow cytometry.

## Conclusions

Human sarcoma specimens yield CD3+ CD8+ TIL which can be expanded *in vitro*, supporting further investigation into the feasibility of adoptive cell transfer as a therapy for these patients. Efforts are currently focused on the scalability of this process and the functional capacity of these TIL. Additionally, the expression of 4-1BB and PD-1 on a substantial of CD3+ CD8+ TIL demonstrates the opportunity to modulate these pathways to improve both yield and function, another endeavor we are presently investigating.

## References

[B1] Pilon-Thomas S Kuhn L Ellwanger S Janssen W Royster E Marzban S Efficacy of adoptive cell transfer of tumor-infiltrating lymphocytes after lymphopenia induction for metastatic melanoma J Immunother 35 8 615 620 22996367 10.1097/CJI.0b013e31826e8f5f PMC4467830

[B2] Turcotte S Gros A Hogan K Tran K Hinrichs CS Wunderlich JR Phenotype and function of T cells infiltrating visceral metastases from gastrointestinal cancers and melanoma: implications for adoptive cell transfer therapy J Immunol 191 5 2217 2225 23904171 10.4049/jimmunol.1300538 PMC3748336

[B3] Tseng W Malu S Zhang M Chen J Sim GC Wei W Analysis of the Intratumoral Adaptive Immune Response in Well Differentiated and Dedifferentiated Retroperitoneal Liposarcoma Sarcoma 2015 547460 25705114 10.1155/2015/547460 PMC4326351

